# Comprehensive Transcriptome Analysis Provides Evidence of Local Thermal Adaptation in Three Loaches (Genus: *Misgurnus*)

**DOI:** 10.3390/ijms17121943

**Published:** 2016-11-24

**Authors:** Shaokui Yi, Sai Wang, Jia Zhong, Weimin Wang

**Affiliations:** 1 Key Lab of Agricultural Animal Genetics, Breeding and Reproduction of Ministry of Education, College of Fisheries, Huazhong Agricultural University, Wuhan 430070, China; yishaokui@foxmail.com (S.Y.); saiwang320@163.com (S.W.); zhongjia@webmail.hzau.edu.cn (J.Z.); 2 Key Lab of Freshwater Animal Breeding, Ministry of Agriculture, College of Fisheries, Huazhong Agricultural University, Wuhan 430070, China

**Keywords:** transcriptome, adaptive evolution, temperature zone, *Misgurnus*

## Abstract

The geographic distribution of three *Misgurnus* species, *M. anguillicaudatus*, *M. bipartitus*, and *M. mohoity*, displays a specific pattern in China, coincident with temperature zones. In this study, we sequenced the transcriptomes of these three species and used the sequences to investigate the lineage-specific adaptations within the genus *Misgurnus*. In total, 51 orphan genes (19 in *M. anguillicaudatus*, 18 in *M. bipartitus*, and 14 in *M. mohoity*) that may contribute to the species-specific adaptations were identified. An analysis of 1392 one-to-one orthologous genes revealed significantly higher ratios of nonsynonymous-to-synonymous substitutions in the *M. mohoity* lineage than in *M. anguillicaudatus*. The genes displaying signatures of positive selection and rapid evolution in *Misgurnus* were involved in four function categories, (1) energy metabolism; (2) signal transduction; (3) membrane; and (4) cell proliferation or apoptosis, implying that these candidate genes play critical roles in the thermal adaptation of the fish to their living environments. We also detected more than five positively selected sites in cldn15lb and isca1, which function as important factors in paracellular Na^+^ transport and Fe/S cluster assembly, respectively. Overall, our study provides valuable insights into the adaptive evolution of loaches from different temperature zones in China and is a foundation for future studies to clarify the genetic basis of temperature adaptation in fishes.

## 1. Introduction

Environmental heterogeneity is one of the most important factors influencing evolutionary trajectories of species. The climate difference between the south (subtropical and tropical zone) and north (warm temperate and mid-temperate zone) of China (Qinling Mountains-Huaihe River line boundary) affects the distributions and environmental adaptations of species, which respond to the temperature variations. The seasonal temperature variations in the northern area (warm temperate zone and mid-temperate zone) are more obvious and rapid than those in the southern area of China. Importantly, the temperature variations in water bodies have immediate effects on fish because the rate of heat exchange between the animal and the ambient water is high. Temperature affects virtually all biochemical, physiological and life history activities of fish [1], and environmental temperature profoundly impacts both the phenotypes and geographical distributions of fish in the southern and northern parts of China. For instance, *Carassius carassius* [2] and *Cyprinus carpio* [3], which can withstand wide seasonal extremes of temperature, overwinter well in ponds in northeast China. In contrast, *Oreochromis niloticus*, which cannot survive temperatures of less than 10–12 °C for more than a few days, is only distributed in southern China [4,5].

Previous studies have reported the biochemical and physiological responses required to adapt to thermal changes, including in muscle fibres [1] and enzymatic activity [6]. Recent studies have primarily used genomic approaches to study the genetic mechanisms of temperature adaptation in fish, and genes involved in physiological adaptations to temperature stress have been identified in many fish species. Gracey et al. [7] performed a large multi-tissue analysis of the responses to increasing cold in the common carp and demonstrated that one response involved the upregulation of transcripts involved in stress responses, cytoskeletal reconstruction, protein catabolism, lipid metabolism and energy pathways. Similar results were observed by Ju et al. [8], who reported the differential gene expression of *Ictalurus punctatus* in response to cold acclimation. Smith et al. [9] conducted RNA-seq analysis in *Melanotaenia fluviatilis* and showed that genes corresponding to critical metabolic pathways were upregulated to ensure temperature tolerance. Although these studies provided important insights into the physiological changes and variability in gene regulation to temperature change, little information is yet available on the evolutionary adaptions of fish species to the seasonal temperature variations in their living environments.

**Figure 1:**
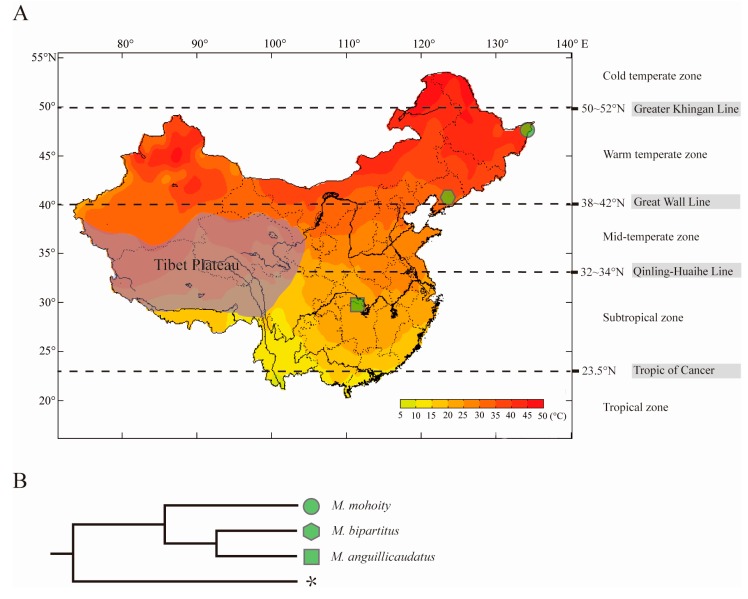
The sampling sites (**A**) and the phylogenetic relationships (**B**) of three species in genus *Misgurnus*. The color gradient in (**A**) indicates annual range of temperature in Mainland China, data from China Meteorological Administration (website: http://data.cma.cn); * in (**B**) indicates the outgroup *Danio rerio*.

In 1925, Nichols [10] first described and reported the species distribution of the genus *Misgurnus* in China. Chen and Zhu [11] proposed that the ancestors of genus *Misgurnus* originated in the Southeast Asia region (north of Wallace’s line), including Kepulauan Sunda Besar, Thailand, Myanmar and south of China (tropical regions), and subsequently radiated into temperate zones via a northern route of expansion, which has been confirmed by Perdices et al. [12,13]. Three species, *Misgurnus anguillicaudatus*, *Misgurnus mohoity*, and *Misgurnus bipartitus*, are distributed throughout China, except on the Tibetan plateau. *Misgurnus anguillicaudatus* is widely distributed in the middle and lower reaches of the Yangtze River Basin, whereas *M. bipartitus* is restricted to north of the Yellow River in China, and *M. mohoity* only occurs in the Amur River Basin in northeast China, Mongolia, and the Far East region of Russia [13,14]. The specific geographical distributions of these three loaches are clearly consistent with the demarcation of temperature zones in China (Figure 1A). Therefore, they provide a rich source of naturally occurring genetic variation within and between species, which can be used to explore biological processes that are important in adaptive evolution. Here, a comprehensive transcriptome analysis of the three species, *M. anguillicaudatus*, *M. bipartitus*, and *M. mohoity*, was undertaken to investigate their evolutionary rates and to identify the genes that may have facilitated the lineage-specific thermal adaptations of these three loach species.

## 2. Results

### 2.1. Transcriptome Assembly and Annotation

In total, 88,842,922, 116,147,180, and 76,502,750 raw reads were generated for *M. anguillicaudatus*, *M. bipartitus* and *M. mohoity*, respectively. After raw data were subjected to quality control to remove and trim the low-quality reads, adapters, polyA tails, and reads containing more than 5% unknown nucleotides, only clean reads were left. With the Trinity de novo assembler, 105,215, 84,887, and 78,707 unigenes were produced for *M. anguillicaudatus*, *M. bipartitus* and *M. mohoity*, respectively. The assembly results are summarized in detail in Table S1. To validate and annotate the assembled unigenes, the unigenes generated with Trinity were subjected to BLASTX searches against public protein databases (NR, Swiss-Prot, KEGG, KOG). The annotation results for the unigenes are summarized in Figure S1. The annotation results for the NR database revealed the majority of the sequences identified with BLAST most closely matched genes from other fish species, as expected, especially *Danio rerio* (65.86%–67.73%), *Astyanax mexicanus* (7.35%–8.06%) and *Larimichthys crocea* (2.83%–3.58%).

### 2.2. Species-Specific Orphan Genes

**Figure 2:**
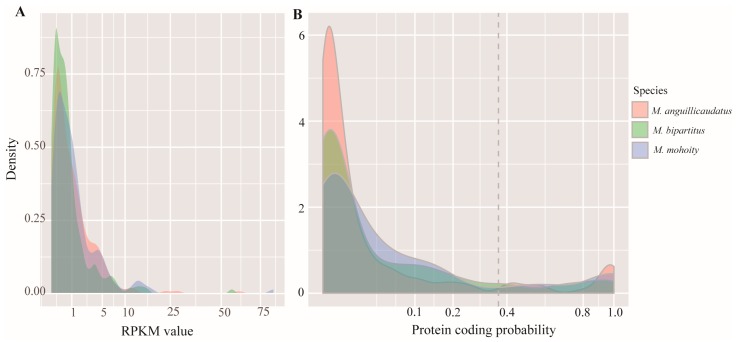
The distribution of RPKM values in species-specific genes (**A**) and protein coding probability of orphan genes predicted by CPAT (**B**) in three loaches. The dotted line in (**B**) represents the 0.364 cut-off for protein coding probability.

Orphan genes, with no recognizable homology to any sequences in other species, may contribute to the species-specific adaptations [15]. To identify orphan genes, a series of BLASTX searches were performed with the three species using an E value threshold of 1 × 10^−5^. The pairwise comparisons of *D. rerio*, *C. carpio*, *O. niloticus* proteomes and the three loach species CDS datasets resulted in a number of species-specific genes. In total, 2545, 1170 and 1005 species-specific genes were identified in *M. anguillicaudatus*, *M. bipartitus* and *M. mohoity*, respectively, after all-to-all BLASTX analyses. It is noteworthy that the average expression levels of these novel genes were low (Figure 2A). Because the assembly accuracy is affected by low read counts, we set a cutoff value of reads per kilobase of transcript per million mapped reads (RPKM > 1) to eliminate the assembly error. To exclude any of these genes that had orthologous in other species, we further checked them against the NR, Swiss-Prot and KEGG databases, after which 181, 163, and 104 orphan genes remained for *M. anguillicaudatus*, *M. bipartitus* and *M. mohoity*, respectively. The coding potential of these novel genes, the prediction of coding potential was performed with the Coding Potential Assessment Tool (CPAT, http://lilab.research.bcm.edu/cpat) [16]. According to the authors of CPAT, the optimum cut-off for protein coding probability was set at 0.364 to extract potential coding genes [17]. From the remaining 448 genes, 51 orphan genes (19 in *M. anguillicaudatus*, 18 in *M. bipartitus*, and 14 in *M. mohoity*) were identified in the genus *Misgurnus* (Figure 2B; Table S2). Because the uniqueness of these genes implies their species specificity, the biological functions of these proteins may encode key determinants of adaptation.

### 2.3. Orthologs Identification and Phylogenetic Relationship

To evaluate the evolutionary dynamics of *Misgurnus* in response to their specific geographic environments, putative single-copy orthologues among three loaches were identified. In total, 1392 one-to-one orthologues, ranging in length from 150 to 5175 bp, were obtained and were used in the subsequent evolutionary analysis. The lengths of the orthologues were reduced when the alignments were trimmed with Gblocks (Figure S2). Phylogenetic trees were inferred from the concatenation of orthologues using NJ and ML methods. The consensus tree revealed that *M. anguillicaudatus* and *M. bipartitus* grouped into one clade with high bootstrap values (100%), implying a close relationship (Figure 1B). This taxonomic relationship is consistent with the results of a previous study based on the mitochondrial genome [18].

### 2.4. Accelerated Evolution in the Misgurnus Lineages

**Figure 3:**
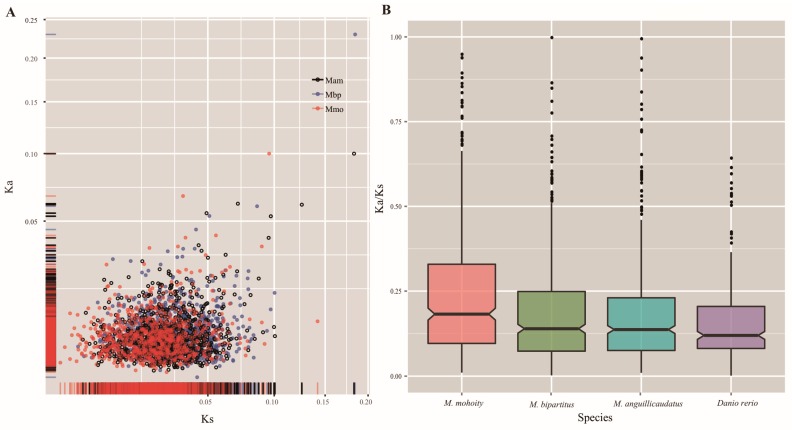
The Ka, Ks, and Ka/Ks ratios for terminal branches were estimated from each orthologous genes in three loaches. (**A**) Scatter plot of Ka and Ks in three loaches based on the free-ratio model; (**B**) The distribution of Ka/Ks ratios for terminal branches. *D. rerio* was selected as a representative. Mam, Mbp, and Mmo in (**A**) indicate *M. anguillicaudatus*, *M. bipartitus* and *M. mohoity*, respectively.

The Ka/Ks ratio is widely recognized as an indicator of selective pressure during evolution [19]. To evaluate the overall differences in the selective constraints at the gene level within the three lineages, the substitution rates (Ka, Ks, and Ka/Ks) for each orthologous gene were calculated using the free ratio model in codeml (Figure 3). The levels of the Ka/Ks ratios showed declining trends in the three lineages, with the *M. mohoity* lineage displaying higher Ka/Ks ratios than the *M. anguillicaudatus* or *M. bipartitus* lineage (Wilcoxon rank sum test, *p* < 3.39 × 10^−7^ or *p* < 9.28 × 10^−8^). The Ka/Ks ratio for each branch of a concatenated alignment of orthologues was also calculated, and the results also indicated that *M. mohoity* had higher Ka/Ks ratios than the *M. bipartitus* and *M. anguillicaudatus* branches. Overall, 254 genes had higher Ka/Ks ratios in the *M. mohoity* lineage than in *M. anguillicaudatus* and *M. bipartitus* lineages, whereas 146 and 143 genes with higher Ka/Ks ratios were observed in *M. anguillicaudatus* and *M. bipartitus*, respectively. To investigate the functions of the lineage-specific genes displaying accelerated evolution (Table S3), the biological functions of the genes with high Ka/Ks ratios were characterized with the KEGG database. Metabolic pathways and the mitogen-activated protein kinase (MAPK) signaling pathway were enriched in all three lineages. The top three functional terms enriched among the genes with high Ka/Ks ratios were “ATP synthase”, “membrane dipeptidase”, and “ribosome biogenesis protein YTM1”, which are associated with energy metabolism and the progress of protein synthesis or hydrolysis in loaches.

### 2.5. Fast Evolving Genes and Positive Selection Analysis

The fast-evolving genes (FEGs), which displayed significantly higher Ka/Ks ratios in a specific lineage compared with the other lineages, were identified using the branch model of codeml. In total, 205, 192, and 191 FEGs were identified in the *M. anguillicaudatus*, *M. bipartitus*, and *M. mohoity* lineages, respectively (Table S4). The branch-site model was used to determine the positively selected genes (PSGs) and the positive selected sites in codons along particular lineages. We identified 63 PSGs in *M. anguillicaudatus*, 53 PSGs in *M. bipartitus*, and 50 PSGs in *M. mohoity* (Table S5). In the comparison of FEGs and PSGs, a set of overlapping genes (*n* = 61) in the sets of PSGs and FSGs was identified (Table 1; Table S6). To identify the potential genes directly involved in adaption evolution of three *Misgurnus* species, we focused on the functions of these overlapping genes. The GO (Gene Ontology) enrichment analysis revealed that these overlapping genes were significantly enriched in “membrane” (*p* = 0.035) and “integral component of membrane” (*p* = 0.033). To further investigate the biological function of these genes, we searched for them manually in the UniProt and NR databases. The functions of the overlapping genes were assigned to four categories (Table 2): (1) energy metabolism (e.g., *dnajb1a*, *ndufs3*, *thrsp*, *srd5a2a*); (2) membrane (e.g., *cldn15lb*, *tm4msf5*, *tmem101*); (3) signal transduction (e.g., *stk17b*, *trim35*, *rad20*); and (4) cell proliferation or regeneration (e.g., *diabloa*, *bmf1*, *cdc23*, *ddx41*).

In the *M. anguillicaudatus* lineage, two genes (*sgcb*, *cldn15lb*) were related to “membrane”, and six genes (*ubiad1*, *ndufb11*, *dnajc19*, *diabloa*, *thrsp*, *alg1*) present in both PSGs and FEGs were involved in “energy metabolism”, suggesting that *M. anguillicaudatus* might have adapted to specific temperature conditions by adjusting its energy metabolism. The *thrsp* gene, which encodes a protein that is important in the regulation of lipid metabolism [20], is rapidly evolving and under positive selection in the *M. anguillicaudatus* lineage. Notably, among the three *Misgurnus* lineages, three genes in the DnaJ (Hsp40) heat shock proteins family were positively selected and fast evolving: *dnajc19* in *M. anguillicaudatus*, *dnajb1a* in *M. bipartitus*, and *dnajc1* in *M. mohoity*. The DnaJ (Hsp40) heat shock protein family, which is involved in Hsp40 chaperone systems by stimulating Hsp70s ATPase activity, is an important group of proteins in the thermal stress responses of fish [21,22]. This result indicates that these *Misgurnus* species induce a group of proteins termed heat shock proteins to adapt to the temperature variations and delay or prevent potential thermal injury [23,24,25].

In summary, (1) at the PSG and FEG levels, the patterns of evolutionary adaptations in the three lineages indicated that *M. anguillicaudatus* is most strongly adapted, followed by *M. bipartitus* then *M. mohoity*; (2) the genes assigned to membrane, signal transduction, energy metabolism and cell proliferation or apoptosis are under positive selection and fast evolving in all three lineages; (3) more genes involved in energy metabolism (e.g., ATP-binding, oxidative phosphorylation, oxidoreductase activity) and signal transduction (e.g., zinc ion binding, G-protein coupled receptor) are enriched in the *M. anguillicaudatus* lineage than in *M. mohoity* or *M. bipartitus*; (4) three DnaJ (Hsp40) genes (*dnajc19*, *dnajb1a*, and *dnajc1*) involved in the response to thermal stress are rapidly evolving and under positive selection in the three lineages, respectively; (5) genes related to the membrane, especially transmembrane protein genes, were positively selected and fast evolving in *M. mohoity*, implying that biological membranes are exposed to stress in the extremely cold temperature of its environment.

### 2.6. Lineage-Specific Positively Selected Sites

**Figure 4:**
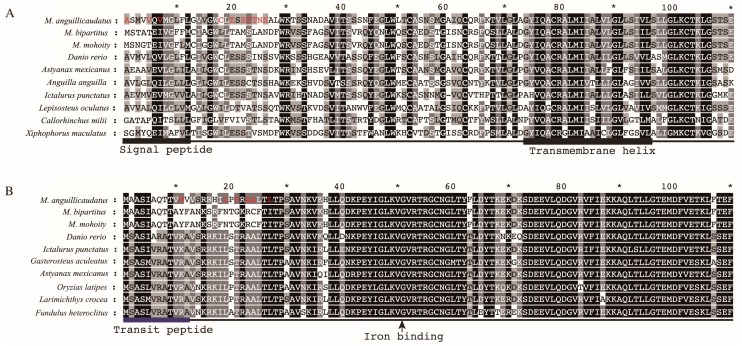
The locations of positively selected sites in Cldn15lb (**A**) and Isca1 (**B**) in *M. anguillicaudatus*. The amino acids colored red represent the positively selected sites. The asterisk above the sequences represents length scale of amino acid sequence.

The positively selected sites were identified using LRTs with FDR correction (*p* < 0.05) and Bayes Empirical Bayes estimates (BEB > 0.95). Twenty, 20, and nine PSGs with sites under positive selection were detected in the *M. anguillicaudatus*, *M. bipartitus*, and *M. mohoity* lineage, respectively (Table S7). The numbers of positively selected sites in the *M. anguillicaudatus* (44 sites) and *M. bipartitus* (32 sites) lineages were higher than that in the *M. mohoity* (13 sites) lineage. Among these positively selective sites, 10 sites in *cldn15lb* and six sites in *isca1*, which are involved in paracellular Na^+^ transport and Fe/S cluster assembly, were detected in the *M. anguillicaudatus* lineage, implying the biological importance of these two genes in adaptations of *M. anguillicaudatus*. Given that no suite temples were available for the homology modeling of Cldn15lb and Isca1 in the public protein databases, the functional significance of the positively selected sites was further investigated by locating them within the secondary structures of Cldn15lb and Isca1, which were predicted with homology searching, SignalP [26], and TMHMM [27]. In Cldn15lb, four positively selected sites (1A, 5V, 7V, 18C) occurred in the signal peptide (Figure 4A), whereas another six sites occurred in chain segments. Similarly, protein structure prediction showed that three positively selected sites of *isca1* occurred in the transit peptide (Figure 4B) and the remained sites were located in the chain segments.

## 3. Discussion

Since the development of next-generation sequencing technology, RNA-seq has been widely used as an efficient and accessible approach to determine the evolutionary histories of non-model organisms when no genome information is available [28,29,30]. Here, we sequenced and assembled the transcriptomes of three closely related *Misgurnus* species, *M. anguillicaudatus*, *M. bipartitus*, and *M. mohoity*, to identify the genetic mechanisms in their adaptation to the temperature variations in their environments and provide valuable information for further studies to understand the genetic makeup of other fishes in thermal adaptations. According to the fishery resources survey undertaken by our laboratory [18] and previous studies [10,13,14,31,32], the geographic distributions of these three *Misgurnus* species show specific patterns, which are coincident with the temperature zones in China. *Misgurnus mohoity* only occurs in Heilongjiang Province (annual range of temperature 50 °C), and *M. bipartitus* is restricted to north of the Yellow River (annual range of temperature 25 °C). The main distribution of *M. anguillicaudatus* is the middle and lower reaches of the Yangtze River Basin (south of Qinling Mountains–Huaihe River Line). Thus, these species present an ideal model in which to investigate the genetic adaptations of fish to different temperature zones.

Orphan genes reflect important evolutionary processes that contribute to evolutionary innovations [33]. In this study, we utilized transcriptome data to identify genes that are present in the three *Misgurnus* species without detectable sequence similarity in the genomes of other organisms. Using strict cutoffs for RPKM and protein coding probability, we identified a total of 51 orphan genes, (19 in *M. anguillicaudatus*, 18 in *M. bipartitus*, and 14 in *M. mohoity*) in the genus *Misgurnus*. The orphan genes may have evolved novel functional roles that contribute to species-specific environmental adaptations [15]. For instance, previous studies reported that the orphan genes in *Daphnia pulex* are specifically activated in response to environmental stimuli [34], and the orphan genes in *Arabidopsis thaliana* are enriched in response to a wide range of abiotic stresses [35]. The orphan genes identified in *Misgurnus* species should be important targets in further studies of the developmental and environmental adaptations of these three closely related species.

In addition to orphan genes, a total of 1392 one-to-one orthologous genes were identified to investigate the adaptive evolution of the three *Misgurnus* lineages. The overall evolutionary rates indicated that the *Misgurnus* lineages exhibit accelerated evolution compared with that of the zebrafish. Among the three *Misgurnus* lineages, the evolutionary rates of *M. mohoity* were highest within the *Misgurnus* lineage, followed by *M. bipartitus* and *M. anguillicaudatus*, suggesting that *M. mohoity* has adaptively accelerated to better adapt to its living conditions. Previous studies have proposed that accelerated evolution can be caused by the relaxation of functional constraints [36], and this requires further investigation with population genomic analyses. To investigate the potential genes directly involved in the adaptive responses to different temperatures, we focused on the overlapping genes of FEGs and PSGs in the three *Misgurnus* lineages. After function annotation of the overlapping genes, they were assigned to four categories: signal transduction, energy metabolism, membrane, and cell proliferation/regeneration. (1) Signal transduction: The transduction of signals indicating abiotic or biotic stress is critical for the adaption and survival of fish under various environment conditions [37]. For example, *oprm1*, a fast-evolving and positively selected gene in *M. anguillicaudatus*, encodes a G-protein coupled receptor that mediates an array of downstream cellular responses to transduce the stimulus signal [38]. Similarly, *rab20* induces small GTPase-mediated signal transduction and promotes the cellular responses to stimulus [39]. Some of these genes related to zinc ion binding, i.e., *trim35*, *gatad1*, *trim63b*, play key roles in detecting and conveying outside signals into cells, allowing them to respond to their living environments in specific ways [40]. Therefore, we infer that zinc ion might be an important molecule in *Misgurnus* in the transmission of temperature stress signaling from the environment; (2) Energy metabolism: Under temperature stress, genes involved in glycolysis (e.g., *alg1*), lipid and steroid metabolism (e.g., *thrsp*, *srd5a2a*), and oxidation–reduction process (e.g., *ubiad1*, *ndufb11*) were enriched, suggesting that fish resist temperature changes with energy metabolism. Previous studies reported that lipid metabolism is associated with cold stress tolerance in common carp [7] and flounder [41] by increasing the level of unsaturated fatty acids and stabilizing lipid fluidity. This indicates that the genes associated with lipid and steroid metabolism mediate the fluidity and flexibility of the cell membranes and are critical for the cellular membrane response to cold stress in loaches; (3) Membrane: Membranes act as selectively protective barriers in organisms and provide a fluid matrix for signal sensing and transduction, molecular transport, and energy metabolism. Transmembrane proteins function as channels and gateways for the transport of specific substances, and temperature is a key factor in the maintenance of the membrane fluidity. To adapt to different environmental temperatures, genes related to transmembrane proteins are fast evolving and positively selected in *M. bipartitus* (*tmem101*) and *M. mohoity* (*tmem88b*, *tmem223*, and *te4sf5*); (4) Cell proliferation or apoptosis: Notably, the cell initiates intracellular apoptotic signaling in response to stress, which may bring about cell suicide. Previous studies proposed that heat, radiation, nutrient deprivation, viral infection, hypoxia, and increased intracellular calcium concentrations can trigger cell apoptosis [42,43,44,45]. The apoptotic process, initiated through the intrinsic pathway or extrinsic pathway, is activated by the intracellular signals generated when cells are stressed. It is noteworthy that genes involved in cell apoptosis were enriched in *M. mohoity*, suggesting that apoptosis has played an important role in the adaptation to low temperatures. As well as the fast-evolving and positively selected genes we identified here, other genes are also important, and will offer further insights into our understanding of the adaptive evolution of the genus *Misgurnus*.

## 4. Materials and Methods

### 4.1. Ethics Statement

All animals and experiments were conducted in accordance with the “Guidelines for Experimental Animals” of the Ministry of Science and Technology (Beijing, China; No. [2006]398, 30 September 2006). All efforts were made to minimize suffering.

### 4.2. Sample Collection

Three loach species, including *M. bipartitus* sampled from the Hun River in Liaoning province (41°17′ N, 125°23′ E), *M. anguillicaudatus* sampled from the Xiliang Lake in Hubei province (29°58′ N, 114°01′ E), and *M. mohoity* sampled from the Amur River in Heilongjiang province (48°21′ N, 135°20′ E), were collected in our study (Figure 1A). All specimens of each species were collected on the same day and under the same conditions. The fishes were euthanized immediately in well-aerated water containing the 100 mg·L^−1^ concentration of tricaine methanesulfonate (MS-222) before tissue collection. Six adult individuals (three males, three females) of each species were selected, and the tissue samples including dorsal muscle, skin, liver, spleen, intestine and brain were immediately collected to extract total RNA. The samples were snap-frozen in liquid nitrogen and stored at −80 °C.

### 4.3. Sequencing, Assembly and Annotation

Total RNA was isolated from tissues using RNAiso Plus Reagent (TaKaRa, Dalian, China) according to the manufacturer’s protocol. RNA quality and quantity was measured using the NanoDrop 2000 spectrophotometer (Thermo Scientific, Wilmington, DE, USA) and Agilent 2100 Bioanalyzer (Agilent, Palo Alto, CA, USA). All the samples were standardized to 500 ng·μL^−1^, and equal volumes of the same tissues from different individuals were combined into one pool. The mRNA was enriched using beads with oligo (dT) and fragmented using fragmentation buffer. Three libraries were sequenced with 125 bp pair end using Illumina HiSeq™ 2500. Raw reads produced from sequencing machines was checked and visualized with FastQC Version 0.11.4 (http://www.bioinformatics.babraham.ac.uk/projects/fastqc/). Transcriptome de novo assembly was performed with Trinity [46]. The CD-HIT-EST [47] was used to remove redundant unigenes with parameter “c = 0.95” and “*n* = 10”. Subsequently, BLASTX alignment (E value ≤ 1 × 10^−5^) to the protein databases (nonredundant [NR], Swiss-Prot, Kyoto Encyclopedia of Genes and Genomes [KEGG], and euKaryotic Orthologous Groups [KOG]) was performed, and the best aligning results are used to determine the direction of unigenes and amino acid sequences. When a unigene happens to be unaligned to none of the above databases, ESTScan software [48] will be introduced to predict its coding region and sequence direction.

### 4.4. Identification of Orthologous Genes

Annotations of coding sequences (CDSs) and proteins of two Cypriniformes and one Perciformes species, including *C. carpio*, *Danio rerio*, and *O. niloticus*, were downloaded from Ensembl database (Release 84). The OrthoMCL algorithm [49] was used to generate core-orthologs for the three whole proteomes datasets. Subsequently, all the putative proteins of the three loach species and core-orthologs were aligned (all against all) using BLASTP, and a score for each pair of proteins with significant matches was assigned (E value ≤ 1 × 10^−7^). Based on the scores, orthologous groups of orthologous genes from different species were identified using OrthoMCL with the default parameters. If more than one unigene were assigned to the same group, the longest unigene was chosen to subsequent analysis. Among the identified groups, only the groups with one to one orthologous relationships were considered for the alignment. PRANK program [50] was used to produce codon alignments for the orthologous sequences with parameter “-codon”. The poor alignment regions were trimmed using Gblocks [51] with the parameter “–t = c”. Alignments with <150 bp (50 codons) were excluded from the dataset. The orphan genes were identified using homology searches method described in previous studies [15,36].

### 4.5. Phylogenetic Analysis and Substitution Rate Estimation

All trimmed nucleotide sequence alignments were concatenated into superalignments for the phylogenetic analyses, with the SCaFoS program [52]. The phylogenetic trees were inferred from the concatenation of 1392 one-to-one orthologous genes (OGs) using the maximum likelihood (ML) and neighbor joining (NJ) methods with 1000 bootstraps, respectively. The ML analysis was performed with RAxML [53] using GTR-GAMMA substitution model, and the NJ tree was inferred with the TreeBeST program (http://treesoft.sourceforge.net/treebest.shtml). The consensus phylogenetic tree was used as the guide tree in the subsequent analysis.

The codeml program in PAML4.5 [54] with the free ratio model (model = 1) was used to estimate the evolutionary rates of the taxa for each orthologous gene and the concatenation of the orthologous genes. The orthologous genes were discarded if N × dN or S × dS < 1 or dS > 1, according to the criterion proposed in a previous studies [28,55]. The evolutionary rates in each lineage were compared using the Wilcoxon rank sum test in R (http://www.R-project.org/). Fast-evolving genes (FEGs) in a specific lineage were identified with the codeml program using the branch model, as described in previous studies [30,36]. The significance of likelihood ratio tests (LRTs) was evaluated to discriminate between alternative models. Multiple testing was corrected with the false discovery rate method (FDR), implemented in R [56]. If the *p* value was < 0.05, and a higher Ka/Ks (the ratio of nonsynonymous to synonymous Substitutions) value was obtained than the foreground branch than the background branches, the gene was considered to be a fast-evolving gene. To identify genes under positive selection, the branch-site model in the codeml program was used, with the null model assuming that all branches have been evolving at the same rate and the alternative model allowing foreground branch to evolve under a different rate [29,30]. As before, LRTs were used to discriminate between alternative models for each gene in the gene set, and *p* value was computed by comparing LRT to the χ^2^ distribution, with the degree of freedom estimated as the difference of parameters between models. We also corrected for multiple testing using the FDR method. When FDR-adjusted *p* value was significant (FDR < 0.05), the Bayes Empirical Bayes (BEB) estimates from each model were then used to identify amino acid sites under positive selection. Gene ontology (GO) enrichment analyses were performed using DAVID Functional Annotation Tool.

## 5. Conclusions

The geographic distribution of three *Misgurnus* species coincident with temperature zones. The orphan genes identified in *Misgurnus* species should be important targets in further studies of the developmental and environmental adaptations of these three closely related species. Meanwhile, the genes displaying signatures of positive selection and rapid evolution in *Misgurnus* were involved in four function categories, including energy metabolism, signal transduction, membrane, and cell proliferation or apoptosis, implying that these candidate genes play critical roles in the thermal adaptation of the fish to their living environments. This result is a prerequisite to revealing the protein function in thermal adaptation. The present study provides some novel insights into the adaptive evolution of loaches from different temperature zones in China and is a foundation for future studies to clarify the genetic basis of temperature adaptation in fishes.

## Figures and Tables

**Table 1:** The summary of estimated Ks, Ka/Ks, GC3, the number of FEGs and PSGs and number of overlapping genes between classes FEGs and PSGs.

Species	Ks	Ka/Ks	GC3	FEGs	PSGs	Overlapping
***M. anguillicaudatus***	0.0127	0.1827	0.5099	205	63	28
***M. bipartitus***	0.0111	0.1994	0.5091	193	53	18
***M. mohoity***	0.0092	0.2271	0.5095	191	50	15

Abbreviation: Ks: substitution rates for synonymous sites; Ka/Ks: the average ratio of nonsynonymous to synonymous Substitutions; GC3: overall GC content in third codon positions; FEGs: fast evolving genes; PSGs: positively selected genes.

**Table 2:** The four function categories of fast evolving and positively selected genes in three *Migurnus* fishes.

Species	Gene	Function Terms	GO Number
**Signal transduction**			
*M. anguillicaudatus* (*n* = 6)	*stk17b*	Intracellular signal transduction	GO: 0035556
	*trim35*	Zinc ion binding	GO: 0008270
	*gatad1*	Zinc ion binding	GO: 0008270
	*oprm1*	G-protein coupled receptor	GO: 0001664
	*selenbp1*	Selenium binding	GO: 0008430
	*card19*	NF-κB signaling	GO: 0043124
*M. bipartitus* (*n* = 3)	*rab20*	GTP binding	GO: 0005525
	*mhc1laa*	Antigen binding	GO: 0006955
	*spred1*	Inactivation of MAPK activity	GO: 0000188
*M. mohoity* (*n* = 2)	*efcc1*	Calcium ion binding	GO: 0005509
	*trim63b*	Zinc ion binding	GO: 0008270
**Membrane**			
*M. anguillicaudatus* (*n* = 2)	*sgcb*	Membrane organization	GO: 0061024
	*cldn15lb*	Tight junction	GO: 0030054
*M. bipartitus* (*n* = 2)	*tmem101*	Transmembrane	GO: 0016021
	*rhbdd2*	Serine-type endopeptidase activity	GO: 0004252
*M. mohoity* (*n* = 4)	*tmem88b*	Integral component of membrane	GO: 0016021
	*tmem223*	Integral component of membrane	GO: 0016021
	*tm4sf5*	Integral component of membrane	GO: 0016021
	*tpbgb*	Integral component of membrane	GO: 0016021
**Energy metabolism**			
*M. anguillicaudatus* (*n* = 6)	*ubiad1*	Antioxidant activity	GO: 0016209
	*ndufb11*	Oxidation-reduction process	GO: 0055114
	*dnajc19*	Hsp70 protein binding	GO: 0030544
	*alg1*	Protein glycosylation	GO: 0006486
	*thrsp*	Lipid metabolic process	GO: 0006629
	*impad1*	Myo-inositol biosynthesis	GO: 0006021
*M. bipartitus* (*n* = 4)	*ndufs3*	Oxidoreductase activity, acting on NAD(P)H	GO: 0004128
	*crot*	Fatty acid beta-oxidation	GO: 0006631
	*pdxp*	Heat shock protein binding	GO: 0031072
	*dnajb1a*	Hsp70 protein binding	GO: 0030544
*M. mohoity* (*n* = 3)	*slc50a1*	Sugar transmembrane transporter activity	GO: 0051119
	*srd5a2a*	Steroid metabolic process	GO: 0008202
	*dnajc1*	ATPase activator activity	GO: 0001671
**Cell proliferation/apoptosis**			
*M. anguillicaudatus* (*n* = 9)	*diabloa*	Apoptotic process	GO: 0006915
	*api5*	Apoptotic process	GO: 0006915
	*fam212aa*	Cartilage development	GO: 0051216
	*yap1*	Cartilage development	GO: 0051216
	*thumpd2*	RNA binding	GO: 0003723
	*bmf1*	Positive regulation of apoptotic process	GO: 0043065
	*lcmt1*	Methyltransferase activity	GO: 0008168
	*ankrd1a*	Sarcomere	GO: 0030017
	*krt222*	Structural molecule activity	GO: 0005198
*M. bipartitus* (*n* = 7)	*gins2*	DNA replication	GO: 0006260
	*cdc23*	Cell division	GO: 0051301
	*plekho1b*	Regulation of myoblast fusion	GO: 1901739
	*cwc25*	Nucleoplasm	GO: 0005654
	*ctdspl3*	Protein dephosphorylation	GO: 0006470
	*ddx41*	Apoptotic process	GO: 0006915
	*ddx52*	rRNA processing	GO: 0006364
*M. mohoity* (*n* = 5)	*ogfr*	Opioid receptor activity	GO: 0004985
	*eepd1*	DNA repair	GO: 0006281
	*dnph1*	Positive regulation of cell growth	GO: 0030307
	*rpz*	Fin development	GO: 0033333
	*cited4b*	Muscle cell differentiation	GO: 0042692

## Supplementary Materials

Supplementary materials can be found at www.mdpi.com/1422-0067/17/11/1943/s1.
