# Supplementary material for: Comprehensive Transcriptome Analysis Provides Evidence of Local Thermal Adaptation in Three Loaches (Genus: Misgurnus)
Source: Int J Mol Sci. 2016 Nov 24;17(12):1943. doi: 10.3390/ijms17121943 (PMC5187763; doi:10.3390/ijms17121943)
Supplement: Supplementary file 1 [file ijms-17-01943-s001.zip › ijms-149387-Supplementary Materials/ijms-149387-supplementary-figure.pdf]

Supplementary Materials: Comprehensive Transcriptome Analysis Provides Evidence of Local Thermal Adaptation in Three Loaches (Genus: *Misgurnus*)

Shaokui Yi, Sai Wang, Jia Zhong and Weimin Wang

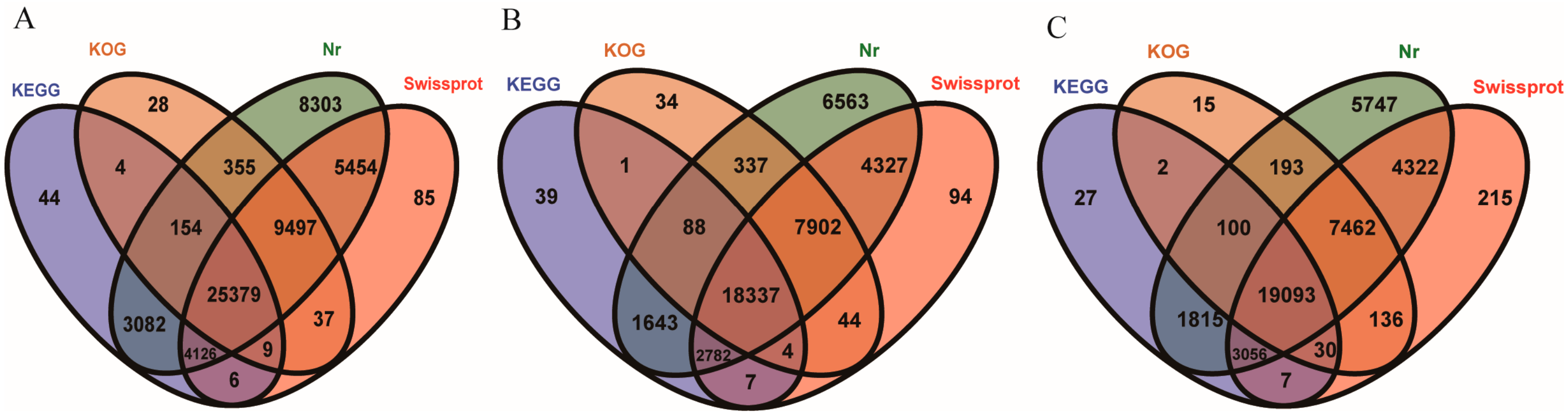

Figure S1. The summary of unigene annotations from four public protein databases in three loaches. (**A**) *M. anguillicaudatus*; (**B**) *M. bipartitus*; (**C**) *M. mohoity*.

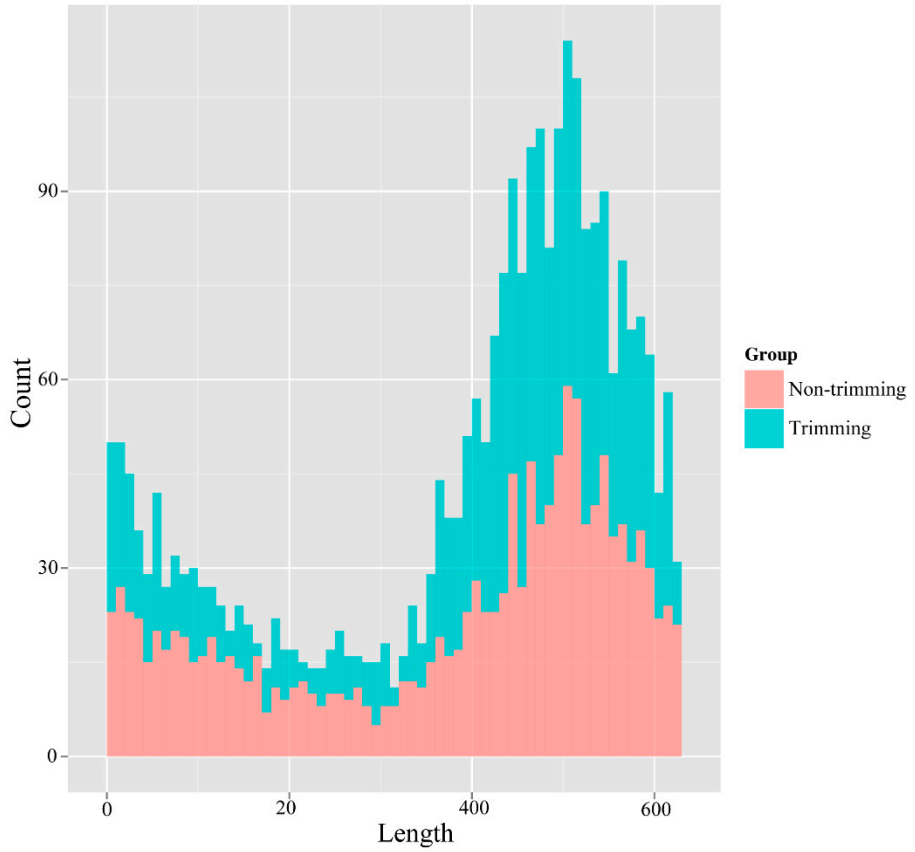

Figure S2. The distribution of alignments length before trimming and after trimming of 1392 orthologous genes in *Misgurnus* species.
